# OATP1A2 mediates Aβ_1-42_ transport and may be a novel target for the treatment of Alzheimer’s disease

**DOI:** 10.3389/fphar.2024.1443789

**Published:** 2024-11-12

**Authors:** Jinhua Wen, Menghua Zhao, Yuwei Xiao, Sihong Li, Weiqiang Hu

**Affiliations:** ^1^ Department of GCP/Psychosomatic Medicine, The First Affiliated Hospital of Nanchang University, Nanchang, China; ^2^ School of Pharmacy, Nanchang University, Nanchang, China

**Keywords:** Alzheimer disease, OATP1A2, beta-amyloid, human astrocyte, drug transporter

## Abstract

Alzheimer’s disease (AD) is a neurodegenerative disease with an unknown cause. Many studies have suggested that the imbalance between the clearance and accumulation of β-amyloid protein (Aβ) in the brain of AD patients is the main cause of AD development of AD. Meanwhile, drug transporters play a key role in the transport of drugs and endogenous substances *in vivo* as well as in the development of many diseases. Could they be related to the imbalance between Aβ clearance and accumulation? OATP1A2 is the most abundant subfamily of organic anion transporting polypeptides (OATPs) that transport amphipathic substrates. Its high bilateral expression in brain endothelial cells suggests it plays a crucial role in delivering drugs and neuroactive peptides to brain tissue. Could it also be involved in mediating the production and accumulation of Aβ in the central system? This could lead to an imbalance between Aβ clearance and accumulation, ultimately resulting in AD development. This hypothesis would be bold and novel in the field of science. In this study, we successfully established the OATP1A2-HEK293T transgenic cell model, and found that the uptake of Aβ_1-42_ by OATP1A2-HEK293T cells was significantly higher than that of NC-HEK293T control cells and human astrocytes by adding different concentrations of Aβ_1-42_ to the cells of each group, suggesting that OATP1A2 expressed in the human brain is involved in Aβ amyloid protein transport.

## 1 Introduction

Alzheimer’s disease (AD) is a neurodegenerative disease of unknown origin that is closely associated with an imbalance in the clearance and buildup of β-amyloid protein (Aβ) in brain tissue. However, the exact mechanism is still unclear (Scheltens et al., 2021). This presents a significant obstacle to the prevention and treatment of AD globally, as there is currently no effective treatment or means to halt its progression. Hence, it is imperative to identify the cause, pathogenesis, and therapeutic targets of AD at the earliest possible stage, as well as to find safe and effective treatment options. This has become an urgent task in AD research.

Further studies suggest that Aβ neurotoxicity is the primary cause of Alzheimer’s disease (AD) development (Hodson, 2018). These studies have confirmed that the deposition of Aβ in the brain can lead to neuronal degeneration and death. In particular, the polysynthetic fibrous form of Aβ has a potent toxic effect on neuronal cells (Tiwari et al., 2019). The human blood-brain barrier (BBB) transporter known as P-glycoprotein (P-gp) is responsible for clearing Aβ from the central nervous system (CNS). However, in AD, the expression of P-gp is downregulated (Storelli et al., 2021; McCormick et al., 2021). P-gp, as an efflux transporter, plays a crucial role in the pathological mechanism of AD and may reduce its incidence and progression, also, other transporters of the ABC family have been shown to affect amyloid efflux from the brain. Are there any uptake transporters involved in the protein uptake of Aβ that worsen the progression of AD? OATPs are significant members of the solute carrier protein family, having a diverse range of substrates that include various endogenous, exogenous, and other substances. In a prior study, we discovered that OATP1B1, which is specifically expressed in the liver, could serve as a crucial “carrier” for transferring Aβ_1-42_ from the blood to the liver (Wen et al., 2021). OATP1B1 may play a role in promoting the metabolism of Aβ_1-42_ by facilitating its entry into liver cells. OATP1A2, the most abundant subfamily of human OATPs, transports amphoteric substrates (Schäfer et al., 2020). Its high expression in endothelial cells on both sides of the brain suggests a critical role in delivering drugs and neuroactive peptides to brain tissue (Schäfer et al., 2020). Could it be involved in mediating the production and accumulation of Aβ in the central system, causing an imbalance between Aβ clearance and accumulation, and leading to the development of AD?

Astrocytes are the most numerous and largest class of neural cells in mammalian brain tissue. It has been extensively shown that astrocytes are involved in the metabolism of Aβ in brain tissue. In the AD brain, it can be observed that astrocytes abnormally aggregate around neural tissues with large aggregates of Aβ amyloid, showing co-localization with amyloid, suggesting a possible scavenging effect of astrocytes on Aβ deposition (Cheng et al., 2020; De Strooper and Karran, 2016; Sidoryk-Wegrzynowicz et al., 2011), but the specific mechanism of how astrocytes are involved in transporting Aβ has been poorly investigated. In this study, we aimed to examine the changes of Aβ_1-42_uptake in HEK293T, OATP1A2-HEK293T transgenic cells, and human astrocytes, to confirm, at the cellular level, whether OATP1A2 is involved in transporting Aβ_1-42_, as well as the expression of OATP1A2 and its transport of Aβ_1-42_ in neuronal cells that are closely related to AD. From the perspective of drug uptake transport, we explored the transport properties of OATP1A2 transporter for Aβ_1-42_ and its potential role in AD disease, with the aim of elucidating the mechanism of imbalance between Aβ protein clearance and accumulation *in vivo*, and providing new targets and therapeutic directions for the treatment of AD disease. We also explored whether OATP1A2 is involved in Aβ_1-42_ uptake and investigated its property of mediating intracellular uptake by constructing the OATP1A2-HEK293T transgenic cell model, to clarify whether there is the expression of OATP1A2 drug transporter in astrocytes and whether it would have a transporter effect on Aβ.

## 2 Methods

### 2.1 Reagents and cell culture

Fetal bovine serum (FBS) (1,122,050) and DMEM high glucose medium (C11965500BT) were purchased from GIBICO (New York, United States), and Tris-Hcl/SDS (1.5 mM, pH 8.8) and Tris-Hcl/SDS (0.5 mM, pH 6.8) were purchased from Shanghai Biotechnology and Biological Engineering Co., Ltd. Next, 10 × PBS (ST476), penicillin-streptomycin solution (100×) (C0222), ECL plus luminescent kit (P0018M), SDS-PAGE protein sample buffer (5×) (P0015L), Western and IP cell lysate (P0013 J), PMSF (ST505), and BCA protein concentration determination kit (P0012)were purchased from Beyotime Institute of Biotechnology (Shanghai, China). Then, 30% acrylamide/bis solution, glycine were purchased from Bio-RAD (California, United States). Aβ_1-42_ (ab2539) was purchased from Abcam Trading Co., Ltd (Shanghai, China). Goat anti-mouse IgG (GAM007) and goat anti-rabbit IgG (GAR0072) and GAPDH (Mab5465) were purchased from Allied Biologicals (Shenzhen, China). HEK293T cells (single clones) and OATP1A2 virus were purchased from Hangzhou HibioTechnology Co., Ltd (Hangzhou, China). Human astrocytes (TX2408) were purchased from Otwo Biotech Inc (ShenZhen, China). Finally, 0.45 μm PVDF membrane wit, DAPI (D9542) and goat anti-rabbit IgG (H + L) Cross-Adsorbed Secondary Antibody (Alexa Fluor 555 Invitrogen™ (A-21428) were purchased from Millipore Inc (Massachusetts, United States), Sigma-Aldrich Trading Co. (Shanghai, China), and Thermo Fisher Scientific Inc (Waltham, Massachusetts, United States), respectively.

HEK293T transgenic cell models stably expressing OATP1A2 were constructed using lentiviral vector technology according to a previous report (Cao et al., 2019). Cells were then cultured in complete medium containing 10% FBS and placed into a 37°C, 5% CO_2_ saturated humidity incubator.

### 2.2 Cellular grouping

NC-HEK293T cells, OATP1A2-HEK293T cells, and human astrocytes were subjected to cell spreading, and different concentrations of exogenous Aβ_1-42_ (0 μM, 0.4 μM, 1 μM, and 2.5 μM, i.e., CK, low, medium, and high concentrations) were added to the cells of each group to observe and detect the accumulation of Aβ_1-42_ in the cells in the groups after incubation for 24, 48, and 72 h, respectively.

### 2.3 Western blot

For Western blotting, 100 μL of Western and IP lysates (PMSF added before use, final concentration 1 mM) were added to the cells in each group, mixed well, and then fully lysed at 4°C for 30 min, centrifuged at 4°C and 12,000 rpm for 15 min. Next, the protein supernatant was collected, separated by 10% SDS- page gel and transferred to PVDF membrane. The PVDF membrane was then immersed in a containment solution containing 5% skimmed milk powder and closed by slow shaking at room temperature for 1 h. The membrane was incubated with the indicated primary antibodies (Aβ_1-42_ diluted 1:1,000, GAPDH diluted 1:5,000) and secondary antibodies (goat anti-mouse IgG, goat anti-rabbit IgG diluted 1:5,000), and then incubated using a The membranes were incubated with the secondary antibodies (goat anti-mouse IgG, goat anti-rabbit IgG dilution ratio of 1:5,000), and then detected using a gel imager (Bio-Rad Laboratories, California, United States, with ChemiDoc XRS + System).

### 2.4 Flow cytometry

Cells from each group were collected uniformly and detected using a flow cytometer from Becton, Dickinson and C (ACCURI C6, New Jersey, United States, the accompanying instrument software was BD Accuri C6 Software). The viral fluorescence intensity was detected by the horizontal FL-1A channel, and the Aβ_1-42_ label fluorescence intensity was detected by the vertical FL-4A channel. The proportion of cells ingesting Aβ_1-42_ in each group was obtained by calculating the proportion of double-positive cells. The relative quantity of cells ingesting Aβ_1-42_ in each group was obtained by calculating the fluorescence intensity of Aβ_1-42_ in double-positive cells.

### 2.5 Laser confocal experiment

A cell plate with good growth status and that had been treated with drug administration was taken, and 1 mL of 4% paraformaldehyde added per well; then the plate was left at room temperature for 20 min to fix the cells. Next, 0.1% Triton X-100 was added to each well, leaving for 20 min to rupture the cell membrane, then rinsed with PBS (3 × 3 min). An appropriate amount of sealing solution (5% BSA pre-prepared in PBS) was added to the coverslip and left to seal for 30 min at room temperature. Then, 100 μL of Aβ_1-42_ (diluted according to the instruction manual) primary antibody was added to the slide and left to stand overnight at 4°C. The cells were then fixed by adding 0.1% Triton X-100 to each well for 20 min to rupture the cell membranes. Alexa Flour secondary antibody (dilution ratio determined according to the instruction manual) was then added dropwise onto the slide and incubated at room temperature under light protection for 1–2 h. Cells were stained with 10 ng/mL DAPI (1:100–1:500) staining solution dropwise under light protection, and incubated at 4°C under light protection for 15–30 min. Finally, cell fluorescence was observed under an A1 laser confocal microscope of Nikon Corporation (Tokyo, Japan) to record and save the pictures.

### 2.6 Statistical analysis

Each of the above experiments was repeated three times for each group, and the data were expressed as mean ± standard deviation (Mean ± SD, n = 3), and the data were saved, and data were processed and plotted using Prism (GraphPad software 9.0 version), and ImageJ (Java 1.8.0, NIH, United States) software. Differences between groups were assessed by one-way analysis of variance (ANOVA) for three or more groups, and statistically analyzed by independent sample Student’s t-test between two groups. *p < 0.05, **p < 0.01, *p* < 0.05 indicated that the differences were statistically significant, and *p* > 0.05 indicated that the differences were not statistically significant.

## 3 Results

**FIGURE 1:**
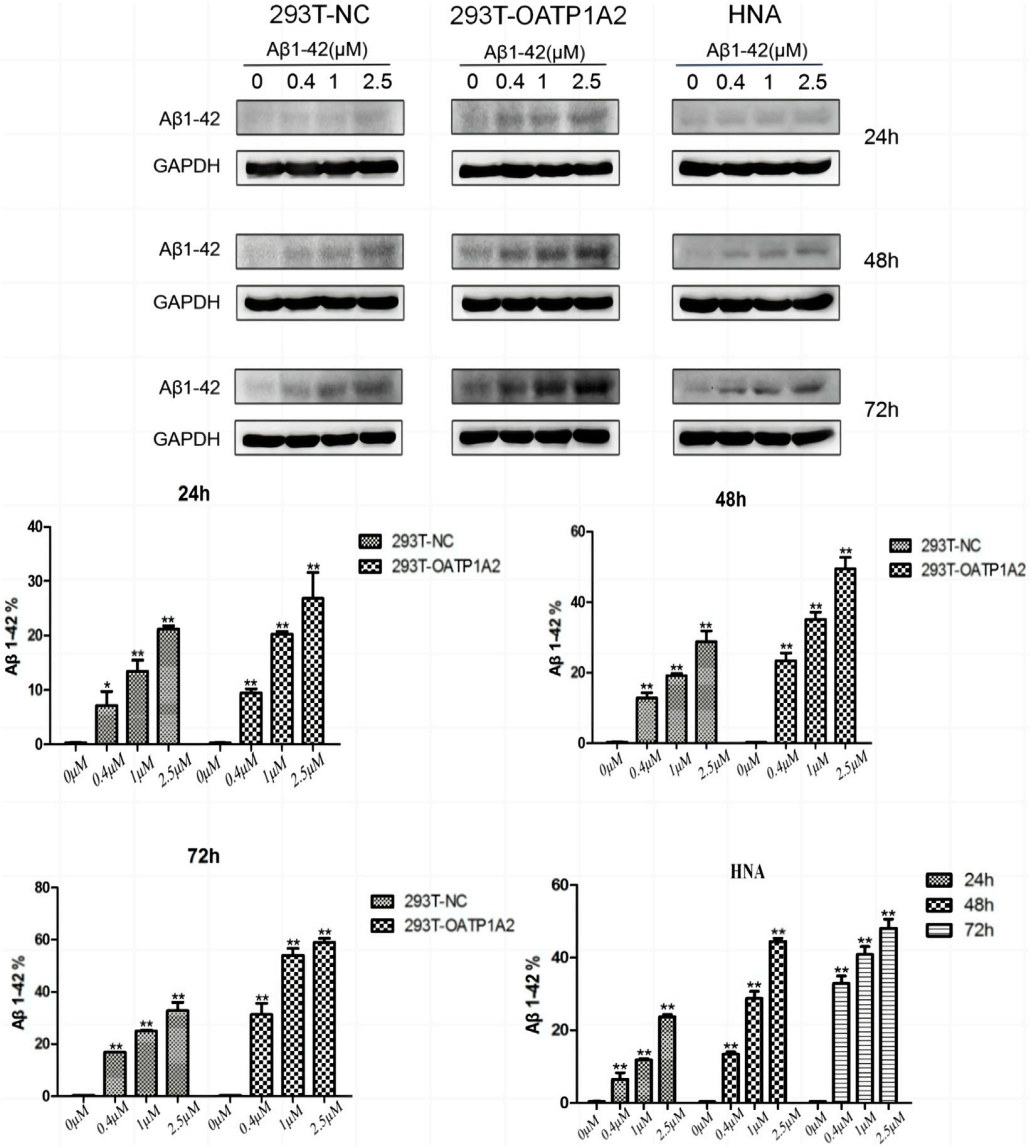
Western blot was used to detect the uptake of amyloid Aβ_1-42_ into cells. The results demonstrated that Aβ_1-42_ protein levels in NC-HEK293T cells, OATP1A2-HEK293T cells, and human astrocytes increased gradually with increasing time (24–72 h) and Aβ_1-42_ concentration (0.4–2.5 μM). This suggests that the uptake of Aβ_1-42_ by these cells is dependent on both time and concentration. Notably, the uptake of Aβ_1-42_ in OATP1A2-HEK293T cells was significantly higher than that in the NC-HEK293T control cells, especially at 48 h and 72 h of culture. At these time points, the uptake of Aβ_1-42_ in OATP1A2-HEK293T cells was nearly twice as high as that in the NC-HEK293T group. Additionally, HNA (human astrocytes) were included in the study.

**FIGURE 2:**
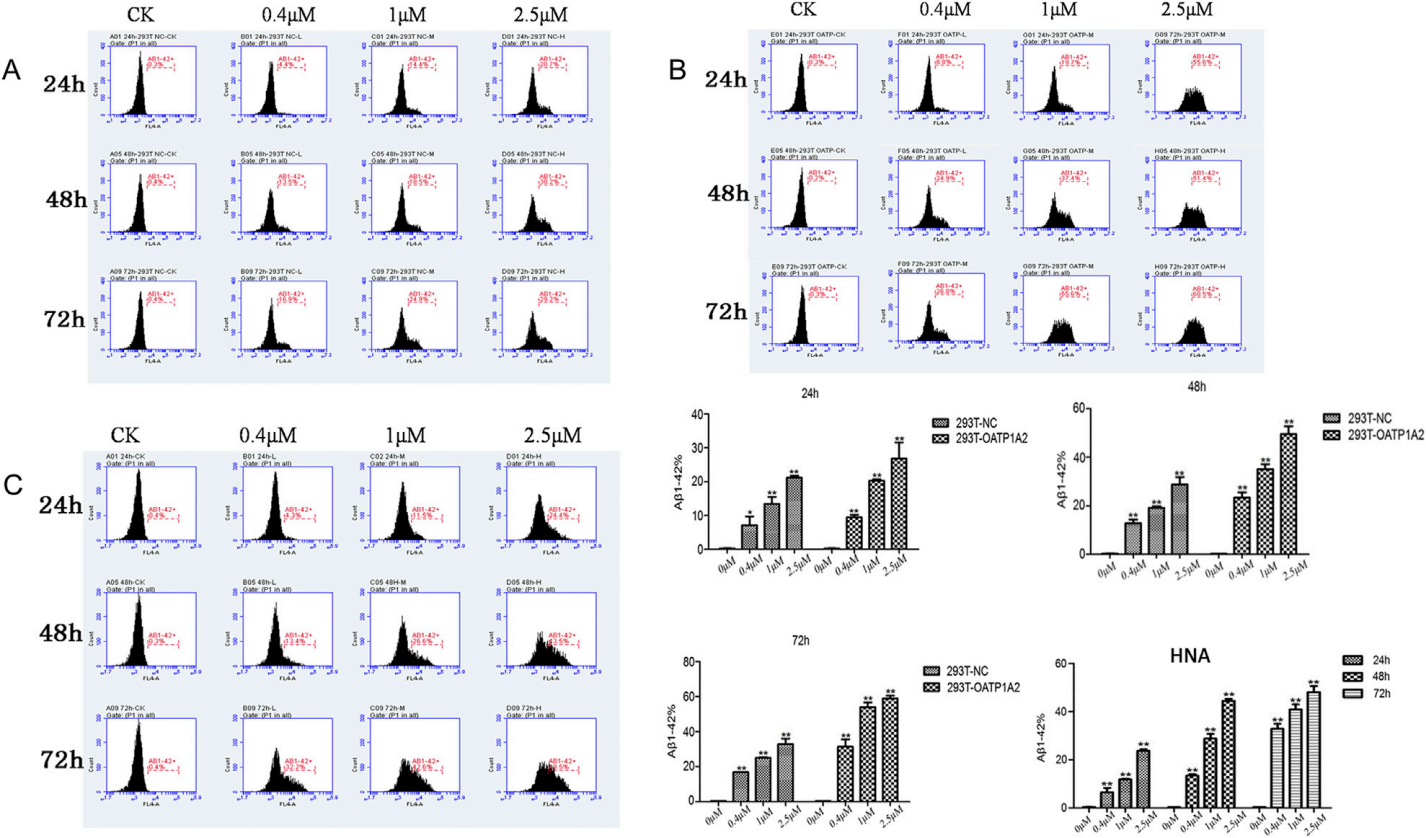
Flow cytometry was used to detect the uptake of amyloid Aβ_1-42_ in three different cell types: 293T-NC cells **(A)**, HEK293T-OATP1A2 cells **(B)**, and human astrocytes **(C)**. The results showed that the uptake of amyloid Aβ_1-42_ in HEK293T-OATP1A2 cells **(B)** was significantly higher than in NC-HEK293T cells after 24, 48, and 72 h of incubation. There was no significant difference in the uptake of amyloid Aβ_1-42_ between human astrocytes and NC-HEK293T cells, but it was significantly higher than the latter at 48 h and 72 h of incubation. HNA: human astrocytes.

**FIGURE 3:**
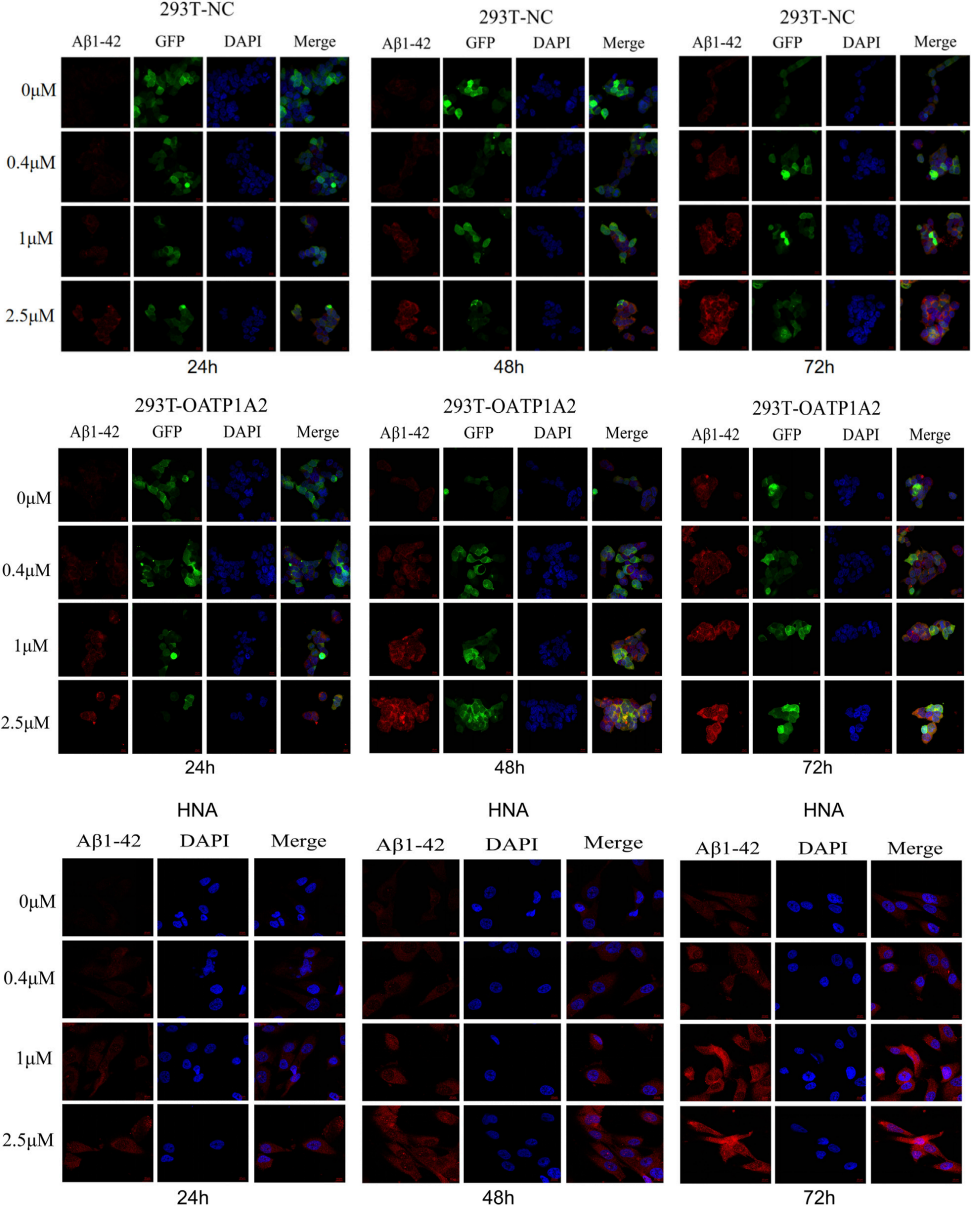
The fluorescence intensity of Aβ_1-42_ gradually increased over time (24–72 h) and concentration (0 μM, 0.4 μM, 1 μM, 2.5 μM) in HEK293T-NC cells, HEK293T-OATP1A2 cells, and human astrocytes. HEK293T-OATP1A2 cells exhibited significantly higher fluorescence intensity of Aβ_1-42_ compared to HEK293T-NC cells under the same conditions. In comparison to human astrocytes, the fluorescence intensity of Aβ_1-42_ in HEK293T-OATP1A2 cells was significantly higher at concentrations of 0.4 μM and 1 μM, whereas at 2.5 μM, the fluorescence intensity was equivalent after 72 h of incubation. HNA: human astrocytes.

In this study, we used lentiviral vector technology to create stable HEK293T transgenic cell models expressing OATP1A2, and the expression of the target gene OATP1A2 mRNA and protein was much higher in the overexpression transgenic group compared to the control cells. After passaging and culturing the overexpressed transgenic cells, we added Aβ_1-42_ at various concentrations (0 μM, 0.4 μM, 1 μM, and 2.5 μM) to HEK293T cells, OATP1A2-HEK293T cells, and human astrocytes for 24 h, 48 h, and 72 h, respectively. The results of Western blot experiments showed that the Aβ_1-42_ protein levels in NC-HEK293T cells, OATP1A2-HEK293T cells, and human astrocytes were gradually increased with the prolongation of time (24–72 h) and the addition of Aβ_1-42_ concentration (0.4–2.5 μM), indicating that the Aβ_1-42_ uptake was time- and concentration-dependent. The uptake of Aβ_1-42_ by OATP1A2-HEK293T cells was significantly higher than that of NC-HEK293T control cells, especially at 48 h and 72 h of incubation, and the uptake of Aβ_1-42_ by OATP1A2-HEK293T was almost twice as much as that of the NC-HEK293T group (Figure 1). The same trend of experimental results appeared in flow cytometry (Figure 2; Table 1) and laser confocal experiments (Figure 3), where the intensity of immunofluorescence of OATP1A2-HEK293T was significantly higher than that of the NC-HEK293T cell group. There was no significant difference in the uptake of amyloid Aβ_1-42_ between human astrocytes and NC-HEK293T cells at 24 h, but the former was significantly higher than the latter at 48 h and 72 h of incubation. These findings suggest that OATP1A2 is involved in the intracellular transport of Aβ_1-42_ and that OATP1A2 is expressed in astrocytes.

**TABLE 1:** Aβ_1-42_ uptake in NC-HEK293 T, OATP1A2-HEK293T and human astrocytes assessed through flow cytometry.

	24 h	48 h	72 h
NC-HEK293T (Aβ_1-42_ 0 μM)	0.30 ± 0.04	0.33 ± 0.09	0.33 ± 0.04
NC-HEK293T (Aβ_1-42_ 0.4 μM)	7.13 ± 2.60**	12.83 ± 1.50**	16.87 ± 0.12**
NC-HEK293T (Aβ_1-42_ 1 μM)	13.45 ± 2.02**	19.15 ± 0.63**	25.02 ± 0.36**
NC-HEK293T (Aβ_1-42_ 2.5 μM)	21.18 ± 0.62**	28.76 ± 3.09**	32.84 ± 3.19**
HEK293T- OATP1A2 (Aβ_1-42_ 0 μM)	0.28 ± 0.03	0.25 ± 0.09	0.27 ± 0.04
HEK293T- OATP1A2 (Aβ_1-42_ 0.4 μM)	9.43 ± 0.77**	23.40 ± 2.17**	31.37 ± 4.27**
HEK293T- OATP1A2 (Aβ_1-42_ 1 μM)	20.27 ± 0.49**	35.11 ± 2.01**	54.01 ± 2.71**
HEK293T- OATP1A2 (Aβ_1-42_ 2.5 μM)	26.83 ± 4.77**	49.41 ± 3.32**	59.00 ± 1.57**
HNA (Aβ_1-42_ 0 μM)	0.41 ± 0.05	0.33 ± 0.05	0.39 ± 0.05
HNA (Aβ_1-42_ 0.4 μM)	6.47 ± 1.86**	13.42 ± 0.58**	32.91 ± 2.06**
HNA (Aβ_1-42_ 1 μM)	11.87 ± 0.31**	28.80 ± 1.93**	40.89 ± 2.17**
HNA (Aβ_1-42_ 2.5 μM)	23.75 ± 0.62**	44.47 ± 0.88**	48.11 ± 2.50**

(Mean ± SD, n = 3, *p < 0.05; **p < 0.01) HNA: human astrocytes.

## 4 Discussion

Studies have shown that the clearance of Aβ from the brain is a complex process mediated by multiple systems and cell types, including transport in blood vessels in the BBB, drainage of lymphatic fluids, and phagocytosis and degradation by microglia as well as immune cells (Wesén et al., 2017; Zurof et al., 2017), which shows that endocytosis may be one of the pathways by which Aβ enters the cells. In the present study, we hypothesized the correlation between the ingestive drug transporter and Aβ. By constructing a transgenic cell model, we verified that Aβ_1-42_ is a transporter substrate of OATP1A2 by using Western blot, cell flow and immunofluorescence, and confirmed that Aβ_1-42_ can be translocated into cytoplasm via the drug transporter. The uptake of Aβ_1-42_ by OATP1A2-HEK293T cells was significantly higher than that of NC-HEK293T control cells, especially at 48 h and 72 h of incubation, and the uptake of Aβ_1-42_ by OATP1A2-HEK293T was almost twice as much as that of the NC-HEK293T group. Meanwhile, the immunofluorescence intensity of A β _1–42_ amyloid protein in OATP1A2-HEK293T cells was significantly higher than that of the NC-HEK293T cell control group. Our findings suggest that the uptake drug transporter of OATP1A2 is involved in mediating the uptake of Aβ_1-42_ in cells *in vivo*, thus triggering the pathological mechanism of AD disease. In addition, our results showed that astrocytes are important regulators of AD pathogenesis and that they can directly interact with amyloid plaques (Itagaki et al., 1989; Arranz and De Strooper, 2019). Indeed, the morphology, physiological function, and activity of astrocytes are altered before Aβ begins to accumulate in the brain to form senile plaques (Dal et al., 2014), and astrocytes also exhibit dual roles in removing and exacerbating Aβ production depending on their state and morphology (De Strooper and Karran, 2016). Therefore, the abnormalities of astrocytes can be used as one of the “warning molecules” to judge the changes in the brain microenvironment of AD. From the experimental results of this paper, the protein expression of OATP1A2 in human astrocytes was higher than that in HEK293T cells, a finding in line with the theoretical basis of high expression of OATP1A2 in brain tissues. This suggests that we can explore the molecular mechanism of astrocytes involved in Aβ transport and clearance from the perspective of drug transporters, which can provide a new target for the study of the pathological process of AD.

In addition, OATP1B1 is a major transporter mediating hepatic transport and uptake of drugs, which is clinically important for the pharmacokinetics and pharmacodynamics of drugs acting in hepatic target organs (Wen et al., 2012; Obaidat et al., 2012). It is worth our special attention that studies have found that the liver participates in the degradation of Aβ and liver function injury can lead to the accumulation of Aβ in the brain tissue of AD patients (Maarouf et al., 2018). In our previous study, we found that the expression of Oatp2 (OATP1B1) was significantly reduced in the liver tissues of AD mice, whereas it was significantly increased in the brain tissues (Wen et al., 2020). In our preliminary experiments, we found that the concentration of Aβ amyloid in OATP1B1-HEK293 transgenic cells was significantly increased after incubation with Aβ amyloid added to the culture medium compared with the HEK293 cell control group, and these findings suggest that OATP1B1 and OATP1A2 may play an important role in the *in vivo* uptake of Aβ protein in the liver and the brain tissues, respectively, and in the transport process. Reduced expression of hepatic OATP1B1 reduces the degradation of Aβ into the liver and leads to an increase in the concentration of Aβ in the circulating blood, and the “uptake” of Aβ in brain tissues exceeds the “discharge” of Aβ when OATP1A2 is highly expressed in brain tissues. At the same time, under the high expression of OATP1A2 in brain tissue, the “intake” of Aβ greatly exceeds the “discharge”, which ultimately leads to an imbalance between the clearance and accumulation of Aβ protein in brain tissue. However, our existing experimental results do not fully demonstrate that astrocytic translocation of Aβ_1-42_ is exclusively mediated by OATP1A2, and further experimental results are needed for verification.

In our future research, we intend to investigate the role of OATPs in AD progression and drug treatment by examining the changes of Aβ_1-42_ expression in blood, liver and brain tissues of AD mice with knockout of Oatp2 or Oatp1a4 genes and drug interventions, and by observing changes in disease symptoms. From the perspective of drug uptake transporters, the present study is of great value to investigate the role of OATPs/Oatps transporters in AD both *in vivo* and *in vitro*, and to provide a new target for AD disease treatment. If this scientific hypothesis is valid, OATPs will become a new target for research on the pathological mechanism of AD and for the development of new drugs and treatment. The present study enriches the study of the transport kinetics of OATPs transporters, expands the connotation of the study of pharmacokinetics, and provides a new mechanism of uptake kinetics for the imbalance of Aβ clearance and accumulation in patients with AD.

## Data Availability

The raw data supporting the conclusions of this article will be made available by the authors, without undue reservation.
