# Correction: OATP1A2 mediates Aβ_1-42_ transport and may be a novel target for the treatment of Alzheimer’s disease

**DOI:** 10.3389/fphar.2026.1903977

**Published:** 2026-07-16

**Authors:** Jinhua Wen, Menghua Zhao, Yuwei Xiao, Sihong Li, Weiqiang Hu

**Affiliations:** 1 Department of GCP/Psychosomatic Medicine, The First Affiliated Hospital of Nanchang University, Nanchang, China; 2 School of Pharmacy, Nanchang University, Nanchang, China

**Keywords:** Alzheimer disease, OATP1A2, beta-amyloid, human astrocyte, drug transporter

There was a mistake in Figure 3 as published. The 48-h immunofluorescence image of 293T-NC in Figure 3 is placed incorrectly. The corrected Figure 3 appears below.

**FIGURE 3:**
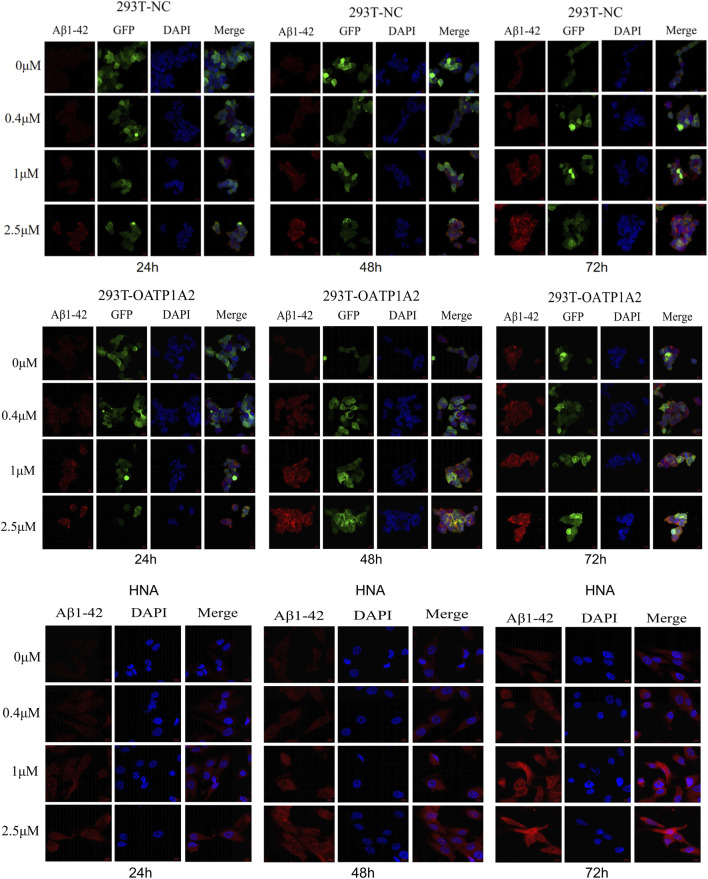
The fluorescence intensity of Aβ_1-42_ gradually increased over time (24–72 h) and concentration (0 μM, 0.4 μM, 1 μM, 2.5 μM) in HEK293T-NC cells, HEK293T-OATP1A2 cells, and human astrocytes. HEK293T-OATP1A2 cells exhibited significantly higher fluorescence intensity of Aβ_1-42_ compared to HEK293T-NC cells under the same conditions. In comparison to human astrocytes, the fluorescence intensity of Aβ_1-42_ in HEK293T-OATP1A2 cells was significantly higher at concentrations of 0.4 μM and 1 μM, whereas at 2.5 μM, the fluorescence intensity was equivalent after 72 h of incubation. HNA: human astrocytes.

The original version of this article has been updated.

